# A Nonrandomized Trial of the Effects of Passive Simulated Jogging on Short-Term Heart Rate Variability in Type 2 Diabetic Subjects

**DOI:** 10.1155/2023/4454396

**Published:** 2023-04-11

**Authors:** Jose A. Adams, Jose R. Lopez, Veronica Banderas, Marvin A. Sackner

**Affiliations:** ^1^Division Neonatology, Mount Sinai Medical Center of Greater Miami, Miami Beach, Florida, USA; ^2^Mount Sinai Medical Center of Greater Miami, Miami Beach, Florida, USA; ^3^Sackner Wellness Products LLC, Miami, Florida, USA

## Abstract

**Background:**

Diabetes mellitus has reached global epidemic proportions, with type 2 diabetes (T2DM) comprising more than 90% of all subjects with diabetes. Cardiovascular autonomic neuropathy (CAN) frequently occurs in T2DM. Heart rate variability (HRV) reflects a neural balance between the sympathetic and parasympathetic autonomic nervous systems (ANS) and a marker of CAN. Reduced HRV has been shown in T2DM and improved by physical activity and exercise. External addition of pulses to the circulation, as accomplished by a passive simulated jogging device (JD), restores HRV in nondiseased sedentary subjects after a single session. We hypothesized that application of JD for a longer period (7 days) might improve HRV in T2DM participants.

**Methods:**

We performed a nonrandomized study on ten T2DM subjects (age range 44-73 yrs) who were recruited and asked to use a physical activity intervention, a passive simulated jogging device (JD) for 7 days. JD moves the feet in a repetitive and alternating manner; the upward movement of the pedal is followed by a downward movement of the forefoot tapping against a semirigid bumper to simulate the tapping of feet against the ground during jogging. Heart rate variability (HRV) analysis was performed using an electrocardiogram in each subject in seated posture on day 1 (baseline, BL), after seven days of JD (JD7), and seven days after discontinuation of JD (Post-JD). Time domain variables were computed, viz., standard deviation of all normal RR intervals (SDNN), standard deviation of the delta of all RR intervals (SD*Δ*NN), and the square root of the mean of the sum of the squares of differences between adjacent RR intervals (RMSSD). Frequency domain measures were determined using a standard Fast Fourier spectral analysis, as well as the parameters of the Poincaré plots (SD1 and SD2).

**Results:**

Seven days of JD significantly increased SDNN, SD*Δ*NN, RMSSD, and both SD1 and SD2 from baseline values. The latter parameters remained increased Post-JD. JD did not modify the frequency domain measures of HRV.

**Conclusion:**

A passive simulated jogging device increased the time domain and Poincaré variables of HRV in T2DM. This intervention provided effortless physical activity as a novel method to harness the beneficial effects of passive physical activity for improving HRV in T2DM subjects.

## 1. Introduction

Diabetes mellitus has reached worldwide epidemic proportions. In 2015, it was estimated that globally 1 in 11 adults aged 20–79 years (415 million adults) had diabetes mellitus, and it is estimated to rise to 642 million by 2040. The global economic burden of diabetes is expected to surpass $2 trillion (US dollars) by 2030 [1]. Ninety percent of diabetes mellitus cases are type 2 diabetes mellitus (T2DM) [2]. Diabetes mellitus is associated with a myriad of end-organ injuries and underlies the severity of illnesses such as coronavirus disease 2019 (COVID-19) and pneumonia with increased mortality and severity of disease [3]. Cardiovascular complications are the leading cause of mortality, morbidity, and disability amongst those with T2DM. Autonomic nervous system (ANS) control of the heart is a complex interaction that involves afferent neuronal impulses transmitted from intrinsic neurons in the heart, extra cardiac intrathoracic ganglia, spinal cord, and brain stem and processed by various parts of the nervous system to regulate neural output to the heart via sympathetic and parasympathetic nerves [4]. Diabetes-associated cardiovascular autonomic neuropathy (CAN) is an underdiagnosed condition in patients with diabetes with a prevalence of 25-75% in patients with T2DM [5]. The pathophysiology of diabetic CAN remains under investigation, but the effects of hyperglycemia and its various biochemical pathways have been shown to be of importance [4, 5].

Heart rate variability (HRV) is a measure of balance within the autonomic nervous system (ANS) of the sympathetic nervous system (SNS) and parasympathetic nervous system (PSN) and reduced HRV as a marker of ANS dysfunction. Recent systematic reviews and meta-analysis showed that T2DM is associated with a decrease in HRV [6, 7]. Physical exercise (mostly aerobic training) has been shown to improve HRV in T2DM [8–10].

Physical activity and exercise remain as two of the pillar interventions in diabetes and have been shown to be positive modulators for improving HRV, whereas the converse (prolonged sitting and sedentary lifestyle) has been shown to reduce HRV [11, 12]. Physical activity has also been shown to play a crucial role in reducing the risks of cardiovascular disease, diabetes, obesity, and other chronic illnesses and its favorable effects linked to the neurohormonal system, particularly in the elderly [13, 14].

We have previously shown that a passive simulated jogging device (JD) which induces pulsations to the forefoot, similarly as locomotion or jogging [15], decreases sedentary time [16, 17], decreases sedentary induced elevation of blood pressure [18], reduces acute hyperglycemic spikes produced by an oral glucose challenge [19], and decreases 24 hr glycemic variability in both T2DM and nondiabetic subjects [16]. A single 30 min session of JD in both seated and supine postures improved heart rate variability in a heterogeneous group of human subjects [20]. We hypothesized that a longer duration (seven days) of JD might improve HRV in T2DM subjects.

## 2. Materials and Methods

### 2.1. IRB Approval

This study and its informed consent forms were approved by the Western Institutional Review Board (WIRB Puyallup, WA 98374-2115), on April 2018 (No. 1184829). The study is registered at ClinicalTrials.gov NCT03550105 (06-08-2018) and conducted between September 2018 and May 2019. This study was part of a larger study that evaluated daily glycemic response, muscle strength, and endurance in healthy volunteers and T2DM. All T2DM subjects were previously diagnosed by their primary care physician as having type 2 diabetes and were currently on insulin and or another medication for control of glycemia. Subjects were recruited by word of mouth from personal contacts. The study protocol was verbally communicated to the subject and provided with the approved written informed consent. All subjects were given the opportunity to ask questions. Interested subjects executed the written informed consent.

### 2.2. Study Participants and Protocol

This study reports the electrocardiogram analysis of HRV of ten ambulatory T2DM individuals. The inclusion criteria in the current protocol were individuals previously diagnosed by their primary physician as having T2DM and currently on treatment, between the ages of 25 and 85 years. The exclusion criteria included inability to provide informed consent, interference with the placement of a continuous glucose-monitoring device (CGM) during the study period, and lack of compliance with the JD protocol. Participants were asked not to drink coffee or caffeinated beverages on any of the study days. Activity level of the participant was determined using the International Physical Activity Questionnaire (IPAQ) short form. The IPAQ scores physical activity levels as low, moderate, or high [21]. The data acquisition was carried out between 8 and 10 a.m. in the seated posture. All subjects received financial remuneration for their participation. On the first study date, a standard two-lead electrocardiogram was placed on the participants and collected for 30 min; the last 20 min was used for analysis (baseline, BL). The participant was instructed in the use of the jogging device (JD) and asked to use the device for at least 90 min per day. Seven days after JD (JD7) usage, the participant returned to the study site for 30 min EKG collection, and the JD was returned. An EKG was repeated seven days after discontinuation of JD usage (Post-JD). All studies were carried out in the seated posture. A study Consolidated Standards of Reporting Trials (CONSORT) flow diagram and checklist are available in the Electronic Supporting Material File (ESM_File_S1).

Body mass index (BMI) was computed to characterize the participants as follows: BMI normal weight—18.5 to 24.9, overweight—25 to 29.9, and obese—30 or more.  Table 1 lists the characteristics of the study participants. Additional participant's demographic and activity information can be found in Table 1S (Electronic Supporting Material File, ESM_File_S1). No attempts were made to recruit any specific subject characteristics; therefore, no attempts were made to control for age, gender, or BMI.

### 2.3. Passive Simulated Jogging Device (JD)

**Figure 1:**
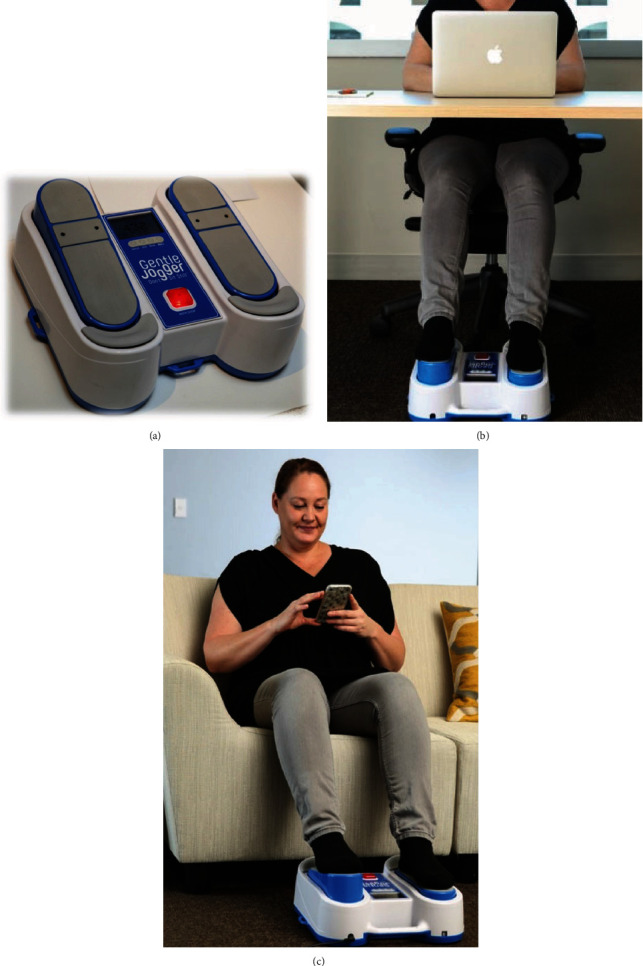
Photographs of the passive simulated jogging device (JD). The photograph depicts a close-up of the JD (a), a seated subject multitasking and working on a desk (b), and a seated subject on a couch (c) with the feet on the passive simulated jogging device (JD).

The portable simulated jogging device (JD) has been previously described [17–20, 22]. The latter incorporates computer-controlled DC motorized movements of foot pedals placed within a chassis to repetitively tap on a semirigid surface to simulate jogging and other locomotion activities while the subject is seated or lying in a bed. It weighs about 4.5 kg with chassis dimensions of 34 × 35 × 10 cm. It is placed on the floor for seated applications and secured on the footplate of a bed for supine application. Its foot pedals rapidly and repetitively alternate between right and left pedal movements to lift the forefoot upward about 2.5 cm, followed by active downward tapping against a semirigid bumper placed within the chassis. In this manner, it simulates the impact of feet impacting against the ground [15]. Each time the foot pedals strike the bumper, a small pulse is added to the circulation. Studies were done at a cadence of ~190 steps/min (Figure 1).

### 2.4. Analysis of HRV

A three-lead electrocardiogram was used for recording of heart rate using a sampling rate of 1000 points per second (LabChart 7 PRO, ADInstruments, Colorado Springs, CO 80906).

The methods used for HRV analysis adhere to the standards developed by the Task Force of the European Society of Cardiology the North A Electrophysiology [23]. RR intervals were obtained from the digitized electrocardiographic signals at a sample rate of 1000 points per sec. The consecutive RR intervals, which were the time intervals between successive pairs of QRS complexes, were detected by using R wave detection (LabChart7 Pro, ADInstruments, Colorado Springs, CO).

HRV was assessed by time and frequency domain methods using three 5 min consecutive RR intervals at baseline (BL), after seven days of JD (JD7), and seven days after discontinuation of JD (Post-JD). All ectopic beats resulting from premature ventricular contraction were removed from electrocardiographic waveforms and missing data replaced by interpolated beats derived from the nearest valid data to analyze the normal RR intervals (NN). The variables used for the time domain analysis include the standard deviation of all normal RR intervals (SDNN), standard deviation of the delta of all RR intervals (SD*Δ*NN), and square root of the mean of the sum of the squares of differences between adjacent NN intervals (RMSSD). Frequency domain measures were determined using a standard Fast Fourier spectral analysis, and the following were calculated on the NN time intervals: very low-frequency power (VLF, 0.01–0.04 Hz), low-frequency power (LF, 0.04–0.15 Hz), and high-frequency power (HF, 0.15-0.4 Hz). LF and HF are reported in normalized units (LFnu and HFnu), representing each power component's relative value in proportion to the total power [24–26].

#### 2.4.1. Poincaŕe Measures

The Poincaré plot is based on the premise that changes in the parasympathetic and sympathetic modulation of the heart rate affect the subsequent RR intervals. Unlike frequency domain measurements, the Poincaré plot analysis is insensitive to changes in trends in the RR intervals [25]. The Poincaré plot provides a scatter of an RR interval plotted against the preceding RR interval as a nonlinear method. The analysis entails fitting an ellipse to the plot, with its center coinciding with the center of the markings. The dispersion of the points, which is perpendicular to the line of identity, measures the width of the Poincaré scattergram and reflects the short-term HRV. The dispersion of points along the line of identity measures the length of the scattergram and indicates the long-term HRV, which reflects the standard deviation of the RR interval (SDNN). The width (SD1) and length (SD2) of the Poincaré determine the width and length of the fitted ellipse to characterize the shape of the plot mathematically [25, 27].

### 2.5. Statistical Analysis

Analyzed EKG data were inputted into STATISTICA (StatSoft Inc., Tulsa, OK) for analysis. The mean values for each parameter were calculated for each epoch and the mean and standard deviation were analysed. In view of the small sample size, each variable was analyzed using the nonparametric Wilcoxon matched-pairs signed rank test, comparing JD7 and Post-JD to BL values. In order to ascertain the effect size of the JD on T2D, we computed the nonparametric common language effect size (CLES) for significant variables. The value represents the probability that a value chosen randomly from JD7 will differ from a value chosen randomly from BL [28, 29]. We performed a post hoc sample size calculation using the primary endpoint of SDNN and using a 30% change this endpoint, with the probability of a type 1 error (*α* = 0.05) and type 2 error (*β* = 0.05); the required sample size of *n* = 7 would be needed to yield a power of 0.95. Statistical analyses were performed using STATISTICA (StatSoft Inc., Tulsa, OK). A *p* value of < 0.001 was considered statistically significant. Data are median and interquartile range (Q1 and Q3), when appropriate data is expressed as mean ± SD as denoted in the text and legends.

## 3. Results

The study group was comprised of ten T2DM participants, who were diagnosed by their primary physician and were on diabetes medications. The study population's age was 58.9 ± 10.7 yrs (mean ± SD) with three male and seven female subjects. Based on BMI, seven of the ten participants were either overweight or obese. Based on the International Society of Global Hypertension Practice guidelines [30], six of the ten participants were hypertensive with two not being treated on initiation of the study. These untreated participants and their primary physicians were alerted to the need for therapy. The age range, BMI, baseline blood pressure, and medications for each subject are listed in Table 1. The mean of the most recent hemoglobin A1c prior to the start of the study was 6.9 ± 0.3%. Self-reported physical activity level was low in nine of the ten participants (Table 1S and Electronic Supporting Material File, ESM_File_S1).

### 3.1. Effects of JD on Heart Rate Variability

**Figure 2:**
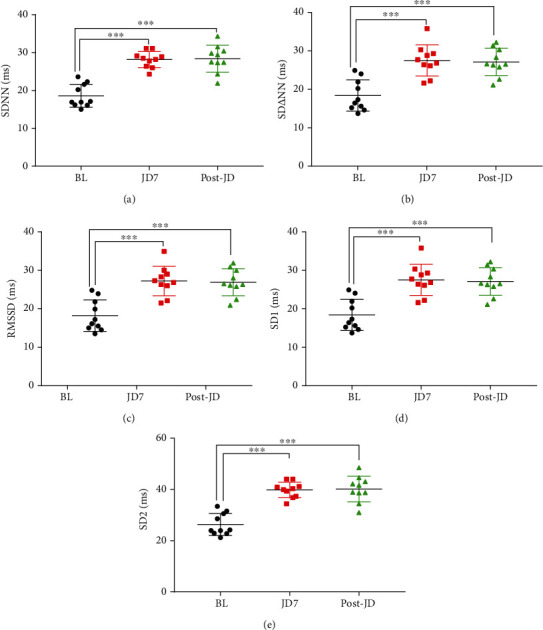
Effects of JD on the linear and Poincaré parameters of HRV. The effects of jogging device (JD) on linear parameters of HRV; (a) standard deviation of all normal RR intervals (SDNN); (b) standard deviation of the delta of all RR intervals (SD*Δ*NN); (c) square root of the mean of the sum of the squares of differences between adjacent NN intervals (RMSSD). The Poincaré parameters of HRV; the mathematical representation of the fitted ellipse of the Poincaré plot. The width (SD1) (e) and length (SD2) of the ellipse (d) at baseline (BL), after seven days of JD (JD7), and seven days after completion of JD (Post-JD). ^∗∗∗^*p* < 0.001, BL vs. JD7 or Post-JD.

Seven days of JD increased the following measures of HRV from baseline, SDNN, SD*Δ*NN, RMSSD, SD1, and SD2, with a carryover effect of at least seven days after JD was discontinued. JD increased SDDN (BL, 17.0 (16.2, 21.6) ms vs. JD7, 28.4 (26.4, 29.1) ms and Post-JD, 28.5 (27.3, 30.4) ms, *p* < 0.001), SD*Δ*NN (BL, 16.8 (15.2, 21.9) ms vs. JD7, 27.3 (26.1, 29.3) ms and Post-JD, 26.6 (25.8, 30.3) ms, *p* < 0.001), and RMSSD (BL, 16.7 (15.0, 21.8) ms vs. JD7, 21.7 (26.0, 29.0) ms, and Post-JD, 26.4 (25.8, 30.0) ms, *p* < 0.001). Similarly, the following Poincaré parameters increased after JD: SD1 (BL, 11.9 (10.8, 15.5) ms vs. JD7, 19.3 (18.5, 20.7) ms and Post-JD, 18.8 (18.2, 21.4) ms, *p* < 0.001) and SD2 (BL, 18.0 (16.2, 21.3) ms vs. JD7, 29.9 (26.0, 32.4) ms and Post-JD, 29.7 (22.7, 34.5) ms, *p* < 0.001) (Figure 2). There were no significant changes in the frequency domain measures of HRV (Table 2).

### 3.2. Effects of Age and Gender on HRV and JD Induced Increase in HRV

Table 2S summarizes the HRV values between young (<59 yrs) and old (>60 yrs), and there were no differences at baseline (BL) between young and old. Both young and old increased the following time domain and Poincaré parameters of HRV from baseline: SDNN, SD*Δ*NN, RMSSD, SD1, and SD2, at JD7 with a carryover effect Post-JD (Table 2S). There was no significant difference between male and female participants (Table 3S) in measures of HRV.

## 4. Discussion

The present study demonstrates that seven days of JD improved the short-term time domain and Poincaré measures of HRV. The effects of JD on HRV measures carried over for at least seven days after discontinuation of JD and are not gender or age dependent (ESM_File_ S1, Table 2S, and T3S).

We compared our data in T2DM for SDNN and RMSSD to the published normative data by Nunan et al. [31] and Shaffer and Ginsberg [25] for short-term HRV in nondiabetic subjects. Our data affirms that both SDNN and RMSSD are markedly reduced in T2DM. Normative data (mean ± SD) for SDNN are 50 ± 16 ms compared to T2DM of 18.6 ± 3.0 ms and RMSSD are 42 ± 15 ms compared to 18.2 ± 4.1 ms. Other investigators have also shown that T2DM subjects have reduced SDNN. Lai et al. also showed that T2DM subjects and specifically those either at risk or with known cardiovascular autonomic neuropathy (CAN) have significantly reduced SDNN compared to those without. In T2DM patients with mild to moderate CAN (data are mean ± SD), SDNN was 17.2 ± 7.7 ms and those with severe CAN 14.2 ± 6.8 ms values which are similar to our T2DM participants at baseline (18.6 ± 3.0 ms) [32]. Furthermore, autonomic dysfunction characterized by vagal depression and sympathetic activity has been shown to start very early, even before the onset of diabetes, and is associated with obesity, hypertension, and metabolic syndrome [33–35]. Cha et al. [36] evaluated the association between HRV and cardiovascular disease in T2DM patients using short-term HRV and followed them over a median of 7.8 years. They found that those with early and definite CAN had lower SDNN and RMSSD. Additionally, time and frequency domain measures of HRV independently predict cardiovascular outcomes in patients with T2DM [36]. The presence of CAN in diabetics has been shown to increase the relative risk of mortality by 3.4 (95% CI: 2.66 to 4.47, *p* < 001) [4] and have higher rate of vasovagal syncope compared to nondiabetics [37]. The pathophysiology of diabetes-related CAN is complex and multifactorial, involving interactions between various factors, such as duration of T2DM, blood pressure, aging-related neuronal death, and hyperglycemia. Hyperglycemia and glycemic fluctuations with induction of reactive oxygen species (ROS) and oxidative stress have been considered the primary cause of CAN [38–40]. Additionally, hyperglycemia-induced ROS has also been shown to damage the microvasculature supplying peripheral nerves [39, 41]. Oxidative stress promoted by acute glycemic excursion (or increased glycemic variability) can induce oxidative damage, toxic advanced glycation products, and activation of nuclear factor kappa beta and protein kinase C pathways, leading to endothelial damage [34]. The Steno-2 trial with strict glycemic control and lifestyle changes in T2DM reduced autonomic neuropathy [38, 41]. Given the multifactorial nature of diabetes-induced CAN, interventions that address many of the pathophysiological causes would be beneficial, including exercise, glycemic control, reduction in oxidative stress, reduced inflammatory phenotype, and improved microvascular flow.

Exercise is one such intervention which improves autonomic function. In T2DM, Bhati et al. [8] have performed a systematic review of exercise training and its effects on cardiac autonomic function. They concluded that exercise training leads to favorable changes in ameliorating autonomic dysfunction as shown by most studies [8]. Benichou et al. [6], using a systematic review and meta-analysis, evaluated the impact of T2DM on HRV parameters. They concluded that T2DM is associated with an overall decrease in HRV, with both sympathetic and parasympathetic decreased activities, which could be explained by the effects of altered glucose metabolism [6].

Most recently, Picard et al. [9] performed a systematic review and meta-analysis on the effects of exercise training on HRV in T2DM. They included twenty-one studies of which nine were randomized. The included exercise interventions were varied and included endurance, resistance, combined endurance resistance, and high interval training. They showed that after exercise training, all HRV parameters increased including SDNN and RMSSD, as well as frequency domain parameters, suggesting that exercise training improves autonomic nervous system activity [9].

Passive simulated jogging (JD) is a method of passive motion of the lower extremities simulating jogging using a motorized pedal device which moves the forefoot in an alternating motion. The upward movement of the pedal is followed by a downward movement of the forefoot tapping against a semirigid bumper to simulate the tapping of feet against the ground; the latter introduces pulsations to the body similar to jogging [15]. Additionally, passive leg movement has been shown to increase microvasculature flow, which is nitric oxide-dependent [42]. Pulsatile shear stress on the vascular endothelium has a plethora of beneficial health effects [22, 43–48]. JD can be used in both seated and supine postures. We previously studied the effects of a single 30 min session of JD in both seated and supine on short-term HRV in a heterogeneous group of adult subjects. JD improved both the time domain and Poincaré parameters of HRV which were irrespective of age and gender [20].

This study was not designed to address the mechanism by which JD modifies HRV; however, several potential mechanisms could account for the observed changes in this T2DM population based on our previous data. Hyperglycemia and large glucose fluctuations are believed to be one of the most important factors in the development of CAN [4, 34]. Strict glycemic control and lifestyle changes in T2DM reduce the development of CAN [5]. Our previous study showed that seven days of JD, in a noncontrolled diet setting, reduces glucose excursions, decreases the 24 hr glucose area under the curve, and reduces glucose variability [16]. Furthermore, in nondiabetic subjects, JD use also blunted the hyperglycemic spikes and areas under the curve induced by an oral glucose challenge [19]. Oxidative stress has been implicated in the pathogenesis of CAN. Using whole-body periodic acceleration (WBPA aka pGz, a predicate device to the JD), we showed that pGz decreases reactive oxygen species in models of increased oxidative stress and increases expression of antioxidant capacity [44, 49–53]. Inflammation also plays a role in CAN. In a cross-sectional design study, Bhati et al. [54] have suggested a pathophysiological link between subclinical inflammation, endothelial dysfunction, and CAN in T2DM. They found that subclinical inflammatory markers such as interleukins (Il-6 and Il-18) and CRP were inversely associated with depressed cardiac vagal and global HRV parameters, while higher NO and eNOS were associated with favorable cardiac vagal activity [54]. In a model of severe inflammatory activation (*E. coli* endotoxin), we showed that WBPA performed before or after induced inflammatory activation, increased survival, and reduced inflammatory activation, including activation of nuclear factor kappa beta [19, 55], reducing the inflammatory phenotype. Microvascular flow has also been implicated in CAN. WBPA has been shown to improve flow-mediated vasodilation (a measure of endothelial function) [56] and improve microvascular flow in animal models [57, 58] and in humans with limb and myocardial ischemia [45, 59].

Nitric oxide (NO) may play a role in the improved HRV observed in this study. Specifically, neuronal nitric oxide synthase (nNOS), which has sympathoinhibitory effects under physiological conditions by acting on different sites of the nervous system, including the paraventricular nucleus, the nucleus of the solitary tract, rostral ventral medulla, the carotid body and nerves in the kidney [60–62]. nNOS inhibition via a selective nNOS inhibitor 1-2 trifluoromethyl imidazole (TRIM) or nNOS knockout mice markedly decreased HRV [63, 64]. There is enhanced vagal control in humans who received NO donor drugs or l-arginine [65]. Additionally, production of nitric oxide via endothelial derived nitric oxide synthase (eNOS), an important vasodilator which increases microvascular flow. eNO has the potential to improve the microvasculature supplying peripheral and central nerves by improving NO bioavailability. JD and WBPA have been shown to increase NO bioavailability [19, 58, 66, 67] and at a cellular level increase both genomic and protein expressions of eNOS and nNOS, via the phospho-inositol 3/protein kinase (Akt) pathway [52, 68]. Based on the multiple targets which JD has been shown to affect, it is a viable, nonpharmacological method to decrease physical inactivity and impact glycemic control and endothelial function [22].

Recently, pharmacological therapy for diabetes with sodium glucose cotransporter type-2 inhibitors (SGLT2i), which have a reasonable safety profile, also shows promise in CAN [69–71]. The proposed mechanism of their effects as both an enhancer of parasympathetic activity and an inhibitor of sympathetic activity has been reviewed [71]. In a diabetic animal model, treatment with SGLT2i ameliorated impaired phosphorylation of Akt and eNOS, restoring eNOS, reducing ROS generation, and decreasing expression of inflammatory markers [72]. Thus, it appears that this pharmacological intervention also targets multiple pathophysiological pathways involved in CAN.

## 5. Study Limitations

There are limitations that must be acknowledged. This study included a small sample of heterogeneous participants in T2DM with ANS dysfunction, with more than half who are also hypertensive and mild to moderately obese; thus, we are unable to control for these confounder variables or medications used. This population is a rather typical patient mix frequently seen in primary care offices. Despite the small sample size, we show a clinically significant effect of 7 days of JD on the various parameters of HRV (SDNN, SD*Δ*NN, RMSSD, SD1, and SD2), with a probability of more than 90% that a value chosen randomly from JD7 differs from a value chosen randomly from BL. We also did not measure additional standardized tests to assess the integrity of the ANS recommended to specifically diagnose CAN [73] and lack additional studies to further characterize additional systemic processes in cardiovascular control. However, the current study population clearly showed reduced HRV compared to normative data and compared to our previous study of JD in a heterogeneous study population [20]. In the previous study, in the seated position at baseline, SDNN was 43 ± 4 ms, RMSSD 36 ± 5 ms, SD1 26 ± 4 ms, and SD2 64 ± 8 ms, in contrast to current T2DM participants in which baseline SDNN was 18.6 ± 3.0 ms, RMSSD 18.2 ± 4.1 ms, SD1 13.0 ± 2.9 ms, and SD2 18.6 ± 3.1 ms [20] (previous data presented as mean ± SD). These striking differences confirm the severity of ANS dysfunction in the current study group. Recently, investigators have shown that the low-frequency component (0.05–0.15 Hz) of the photoplethysmography (PPG) signal reflects the autonomic control of blood pressure [74]. PPG is a readily accessible and wearable technology (in contrast to ECG) that may be used to monitor various cardiovascular parameters in health and disease such as oxygen saturation, heart rate, blood pressure, respiration, endothelial function, vascular and microvascular flow, and autonomic function [75–80]. The use and analysis of the PPG signal in this study may have provided further insight. In the present study, we used linear time domain-based HRV analysis; other investigators have utilized nonlinear HRV analysis methods, based on chaos theory such as analysis of trend fluctuations, correlation function, exponent of Hurst, fractal dimension, and the exponent of Lyapunov; these have also been reported using additional PPG [81]. These methods may prove to be useful in further research on HRV. Finally, we also did not study shorter or extended use of JD (more than 7 days), due to the limited availability of equipment.

## 6. Conclusions

The present study demonstrates that seven days of JD in T2DM improves the short-term time domain and Poincaré measures of HRV. The effects were not age- or gender-dependent and appear to last at least for seven days after discontinuation of JD. These data, coupled with our previous findings in T2DM subjects, support the early application of JD along with pharmacologic, behavioral, and lifestyle modifications in the therapeutic armamentarium for T2DM. Exercise remains a critical and important therapeutic intervention in diabetes. JD is not a surrogate for exercise but a simple, effortless modality to decrease physical inactivity since it can be used during sedentary activities of work and a welcomed intervention for those who can not exercise due to physical and cognitive limitations or have a prolonged work sitting environment.

## Figures and Tables

**Table 1:** Study participants' characteristics.

Subject	Gender	Age range (yrs)^∗^	BMI	Blood pressure (*S*/*D*) (mmHg)	Medications
1	F	73-75	27.8^ow^	146/82	Atorvastatin, losartan, Synthroid, Jentadueto, clonazepam, Brilinta, famotidine, Wellbutrin, and pantoprazole
2	F	64-66	44.8^ob^	134/81	Tradjenta, metformin
3	F	58-63	29.3^ow^	143/83	Lisinopril, B complex, metformin, and insulin
4	F	51-56	28.8^ow^	161/96	Metformin, insulin
5	F	76-80	29.0^ow^	152/76	Synthroid, Vitoza
6	M	66-70	24^nl^	140/80	Insulin, potassium, atorvastatin, metoprolol, lisinopril, amlodipine, pantoprazole, clopidogrel, and bumetanide
7	M	62-65	29.7^ow^	130/71	Metformin
8	M	44-49	24^nl^	135/75	Insulin, levothyroxine, vitamin B12, Truvada, and magnesium
9	F	44-49	26.2^nl^	126/80	Glipizide, gemfibrozil, metformin, and aspirin
10	F	51-56	29.7^ow^	142/83	Metformin
	M = 3 F = 7	58.9 ± 10.7	29.4 ± 5.5		

This table represents the study participants characteristics: study subject number (No), gender, age range (years), calculated body mass index (BMI) (nl: normal weight; ow: overweight; ob: obese), baseline blood pressure (systolic/diastolic), and current medications. Mean and standard deviation (SD) for each column. ^∗^Age range is used to protect any participant identifiable information.

**Table 2:** Effects of JD on parameters of heart rate variability.

	BL	JD7	Post-JD	Effect size of JD CLES
Heart rate (BPM)	77.8 (70.4, 81.9)	77.2 (68.6, 84.0)	78.7 (74.4, 81.4)	
SDNN (ms)	17.0 (16.2, 21.6)	28.4 (26.4, 29.1)^∗^	28.5 (27.3, 30.4)^∗^	92.3%
SD*Δ*NN (ms)	16.8 (15.2, 21.9)	27.3 (26.1, 29.3)^∗^	26.6 (25.8, 30.3)^∗^	94.2%
RMSSD (ms)	16.7 (15.0, 21.8)	21.7 (26.0, 29.0)^∗^	26.4 (25.8, 30.0)^∗^	94.6%
SD1 (ms)	11.9 (10.8, 15.5)	19.3 (18.5, 20.7)^∗^	18.8 (18.2, 21.4)^∗^	94.1%
SD2 (ms)	18.0 (16.2, 21.3)	29.9 (26.0, 32.4)^∗^	29.7 (22.7, 34.5)^∗^	92.3%
LFnu	39.7 (27.1, 52.8)	51.4 (40.5, 81.5)	56.5 (24.9, 65.8)	
HFnu	41.6 (24.0, 53.4)	26.1 (14.0, 47.4)	31.5 (20.3, 40.6)	
LF/HF	0.9 (0.5, 2.2)	1.5 (0.9, 5.8)	1.5 (0.9, 3.6)	

Parameters of heart rate variability at baseline (BL), after 7 days of the jogging device (JD7), and 7 days after discontinuation of JD (Post-JD). Heart rate (beats per minute (BPM), standard deviation of all normal RR intervals (SDNN), standard deviation of the delta of all RR intervals (SD*Δ*NN), and square root of the mean of the sum of the squares of differences between adjacent NN intervals (RMSSD). The mathematical representation of the fitted ellipse of the Poincaré plot. The width (SD1) and length (SD2) of the ellipse. Frequency domain parameters determined using a standard Fast Fourier spectral analysis calculated on the NN time intervals; low-frequency (LF) power and high-frequency (HF) power are reported in normalized units (LFnu and HFnu). The last column represents the effect size of JD on the various statistically significant parameters, determined using common language effect size (CLES) comparing BL to JD7. The CLES values represent the probability that a value chosen randomly from JD7 will differ from a value chosen randomly from BL. Median and interquartile (Q1 and Q3) for each. ^∗^BL vs. JD7 or Post-JD, *p* < 0.001.

## Data Availability

The datasets generated during and/or analyzed during the current study are available from the corresponding author on reasonable request.

## Supplementary Materials

Supplementary Materials ESM_File _S1: CONSORT checklist, study diagram, and tables. This file contains the Consolidated Standards of Reporting Trials (CONSORT) checklist and a study diagram. Table 1S contains a summary of additional study participant demographics, physical activity levels, and hemoglobin A1c. Tables 2S and 3S are the effects of age and gender on the heart rate variability changes induced by the jogging device (JD). Table 4S shows the differences between 7 days of jogging device (JD7) and 7 days after completion of JD (Post-JD) and baseline (BL) for parameters of the HRV for the entire study group.
