# Supplementary material for: A Nonrandomized Trial of the Effects of Passive Simulated Jogging on Short-Term Heart Rate Variability in Type 2 Diabetic Subjects
Source: J Diabetes Res. 2023 Apr 11;2023:4454396. doi: 10.1155/2023/4454396 (PMC10113059; doi:10.1155/2023/4454396)
Supplement: Supplementary Materials — ESM_File _S1: CONSORT checklist, study diagram, and tables. This file contains the Consolidated Standards of Reporting Trials (CONSORT) checklist and a study diagram. Table 1S contains a summary of additional study participant demographics, physical activity levels, and hemoglobin A1c. Tables 2S and 3S are the effects of age and gender on the heart rate variability changes induced by the jogging device (JD). Table 4S shows the differences between 7 days of jogging device (JD7) and 7 days after completion of JD (Post-JD) and baseline (BL) for parameters of the HRV for the entire study group. [file 4454396.f1.zip › Supplemental Data Files JD on HRV Diabetes 8.24.22 docx.pdf]

## ***Supplemental Data Files***

***Table 1S*** Summary of Additional Study Participant Demographic, Physical Activity Level and Hemoglobin A1c.

| ***Race Ethnic Group*** | **Number of Participants** | **% of Study Group** |
|---|---|---|
| Non-Hispanic Whites | 3 | 30 |
| Non-Hispanic Blacks | 0 | 0 |
| Asians/South Pacific Islanders | 0 | 0 |
| Hispanic | 7 | 70 |
| ***Hispanic Distribution*** | | |
| Central and South American | 2 | 29 |
| Cuban | 4 | 57 |
| Mexican | 0 | 0 |
| Puerto Rico | 1 | 14 |
| ***Physical Activity Level (PAL)*** | **Mean** | **SD** |
| MET-min/week VPA | 0 | 0 |
| MET-min/week MPA | 16 | 51 |
| MET-min/week walking | 318 | 133 |
| MET-min/week total | 334 | 144 |
| Minutes/week sitting time | 420 | 85 |

PAL* 1= Moderate 9=LOW

| Hemoglobin A1c (%) | Mean | SD |
|---|---|---|
| Study Group | 6.9 | 0.3 |
| Male (n=3) | 7.1 | 0.1 |
| Females ( n=7) | 6.8 | 0.3 |

*Legend: Tale 1S*

*This table represents an additional summary of the study participants; race/ethnic status, physical activity level (PAL) based on the categorical groups outlined in IPAQ Short Form Questionnaire, and the most recent hemoglobin A1c level prior to enrolling in the study. Time variables for minutes per week converted to MET (Metabolic Equivalent) for vigorous (MET-min/week VPA), Moderate (MET-min/week MPA), walking (MET-min/week walking), sum of the aforementioned (MET-min/week total) and minutes per week of sitting time. Mean and standard deviation (SD) for these. * The IPAQ scores physical activity levels (PAL) as; Low, Moderate, or High (C. L. Craig, Marshall, A. L., Sjostrom, M., et al., "International physical activity questionnaire: 12-country reliability and validity," Medicine and science in sports and exercise, 35, 8, 1381-95, 200). The scoring protocol for IPAQ utilized the file created by Andrea Di Blasio, Francesco Di Donato and Christian Mazzocco at the Endocrinology Unit, Department of Medicine and Aging Sciences, G. d'Annunzio University of Chieti-Pescara (Italy), which can be found* https://sites.google.com/site/theipaq/scoring-protocol

*Table 2S.* **The Effects of Age on JD Induced Changes in HRV**

| | BL | | JD7 | | Post-JD | |
|---|---|---|---|---|---|---|
| | **Young** | **Old** | **Young** | **Old** | **Young** | **Old** |
| **Heart Rate (BPM)** | 74.1(7.0) | 80.9(7.9) | 71.5(6.7) | 82.7(8.9) | 75.5(6.0) | 78.9(10.2) |
| **SDNN(ms)** | 20.0(3.1) | 17.2(2.6) | 27.8(2.0)[a] | 28.5(2.5)[b] | 27.7(4.9)[a] | 29.1(1.8)[b] |
| **SDΔNN(ms)** | 20.1(4.4) | 16.6(3.2) | 29.9(3.6)[a] | 25.1(3.1)[b] | 26.2(4.6)[a] | 28.0(2.5)[b] |
| **RMSSD(ms)** | 19.9(4.4) | 16.5(3.3) | 29.5(3.3)[a] | 24.9(3.0)[b] | 26.0(4.4)[a] | 27.8(2.4)[b] |
| **SD1(ms)** | 14.2(3.1) | 11.8(2,3) | 21.2(2.6)[a] | 17.7(2.2)[b] | 18.6(3.2)[a] | 19.8(1.8)[b] |
| **SD2(ms)** | 19.5(3.6) | 17.7(2.5) | 24.8(6.3)[a] | 31.4(3.6)[b] | 27.8(10.7)[a] | 29.8(5.4)[b] |
| **LFnu** | 46.2(27.1) | 42.8(20.8) | 50.6(24.9) | 61.4(22.3) | 52.1(22.5) | 45.8(27.7) |
| **HFnu** | 31.9(18.2) | 45.5(18.2) | 34.0(21.3) | 27.8(18.2) | 27.1(9.5) | 40.6(25.6) |
| **LF/HF** | 2.9(3.7) | 1.4(1.6) | 2.5(2.4) | 4.1(4.1) | 2.6(2.7) | 2.2(2.3) |

*Legend*: Table 2S

The effects of age on HRV parameters for Older (subjects >59 yrs, n=5) and Younger ( subjects < 59 yrs, n=5). Standard deviation of all normal RR intervals (SDNN), standard deviation of the delta of all RR intervals (SDΔNN), square root of the mean of the sum of the squares of differences between adjacent NN intervals (RMSSD). Poincare parameters of SD1 and SD2, Frequency domain parameters determined using a standard Fast Fourier spectral analysis calculated on the NN time intervals; low-frequency power (LF), high-frequency power (HF) LF, and HF powers are reported in normalized units (LFnu, and HFnu). Baseline (BL) after 7 days of Jogging Device (JD7) and 7 days after completion of JD (Post-JD). In Younger subjects [a]= BL vs. JD7 or Post-JD $p< 0.01$. In Older subjects [b]= BL vs. D7 or Post-JD $p< 0.01$. ***Data are Mean(SD)***

*Table 3S.* **The Effects of Gender on JD Induced Changes in HRV**

| | **BL** | | **JD7** | | **Post-JD** | |
|---|---|---|---|---|---|---|
| | **Female** | **Male** | **Female** | **Male** | **Female** | **Male** |
| **Heart Rate (BPM)** | 77.5(8.4) | 77.5(8.1) | 76.2(9.4) | 79.2(11.4) | 77.3(5.7) | 77.0(14.1) |
| **SDNN(ms)** | 19.7(3.0) | 16.0(1.0) | 28.3(1.7) | 27.7(3.4) | 28.9(3.9) | 27.1(2.7) |
| **SDΔNN(ms)** | 19.9(3.9) | 14.9(1.4) | 28.1(4.2) | 26.2(4.1) | 26.6(4.1) | 28.4(1.9) |
| **RMSSD(ms)** | 19.7(4.0) | 14.7(1.3) | 27.7(4.0 | 26.0(3.9) | 26.4(4.0) | 28.1(1.8) |
| **SD1(ms)** | 14.1(2.8) | 10.5(1.0) | 19.8(3.0) | 18.5(2.9) | 18.8(2.9) | 20/1(1.3) |
| **SD2(ms)** | 19.3(3.5) | 17.1(0.8) | 28.1(4.2) | 28.0(10.3) | 30.4(8.2) | 25.2(7.9) |
| **LFnu** | 40.7(22.8) | 53.4(24.8) | 56.4(21.8) | 55.2(30.9) | 52,6(23.5) | 40.5(28.1) |
| **HFnu** | 39.8(16.9) | 36.1(26.2) | 32.7(20.6) | 26.7(17.6) | 31.0(15.9) | 40.7(29.3) |
| **LF/HF** | 2.1(3.2) | 2.3(1.9) | 2,9(2.6) | 4.1(5.2) | 2.8(2.7) | 1.6(1.7) |

*Legend**: Table 3S***

The effects of gender on HRV parameters for Males (n=3) and Females (n=7). Standard deviation of all normal RR intervals (SDNN), standard deviation of the delta of all RR intervals (SDΔNN), square root of the mean of the sum of the squares of differences between adjacent NN intervals (RMSSD). Poincare parameters of SD1 and SD2, Frequency domain parameters determined using a standard Fast Fourier spectral analysis calculated on the NN time intervals; low-frequency power (LF), high-frequency power (HF) LF, and HF powers are reported in normalized units (LFnu, and HFnu) Baseline (BL) after 7 days of Jogging Device (JD7) and 7 days after completion of JD (Post-JD). There were no statistically significant differences between males and females at BL ***Data are Mean (SD)***

*Table 4S Differences (delta after-before) for HRV parameters for the entire group*

| | **Δ (JD7-BL)** | **Δ (Post-JD-BL)** |
|---|---|---|
| **Heart Rate (BPM)** | -1.6(-7.2,5.0) | 1.0(-2.9,4.5) |
| **SDNN(ms)** | 9.0(6.8,12.8) | 10.7(7.4,13.3) |
| **SDΔNN(ms)** | 11.35(3.7,13.9) | 9.9(4.3,13.9) |
| **RMSSD(ms)** | 11.2(3.5,13.8) | 10.1(4.3,13.8) |
| **SD1(ms)** | 8.0(2.6, 9.9) | 7.0 (3.1,9.9) |
| **SD2(ms)** | 8.5(7.5,16.3) | 12.6(3.1,16.0) |
| **LFnu** | 10.2(-0.4,25.2) | 1.2(-22.2,29.9) |
| **HFnu** | -6.8(-22.3,4.8) | -5.3(-15.4,4.6) |
| **LF/HF** | 0.5(-0.8,3.5) | 0.2(-0.7,1.3) |

*Legend**: Table 4S***

Differences between 7 days of Jogging Device (JD7) and 7 days after completion of JD (Post-JD) and baseline (BL) for parameters of the HRV for the entire study group. Standard deviation of all normal RR intervals (SDNN), standard deviation of the delta of all RR intervals (SDΔNN), square root of the mean of the sum of the squares of differences between adjacent NN intervals (RMSSD). Poincare parameters of SD1 and SD2, Frequency domain parameters determined using a standard Fast Fourier spectral analysis calculated on the NN time intervals; low-frequency power (LF), high-frequency power (HF) LF, and HF powers are reported in normalized units (LFnu, and HFnu). ***Data are Median and IQR (Q1,Q3)***

***Figure 1S. CONSORT FLOW DIAGRAM***

*Legend*: Eleven participants were enrolled all diagnosed as type 2 diabetics by their primary care physician and on either insulin or other medications to control glucose. Since the study was designed to mimic real world situation, no attempts were made to exclude any subjects. One participant was withdrawn due to lack of compliance with the usage of passive simulated jogging device (JD).

Figure 1 S

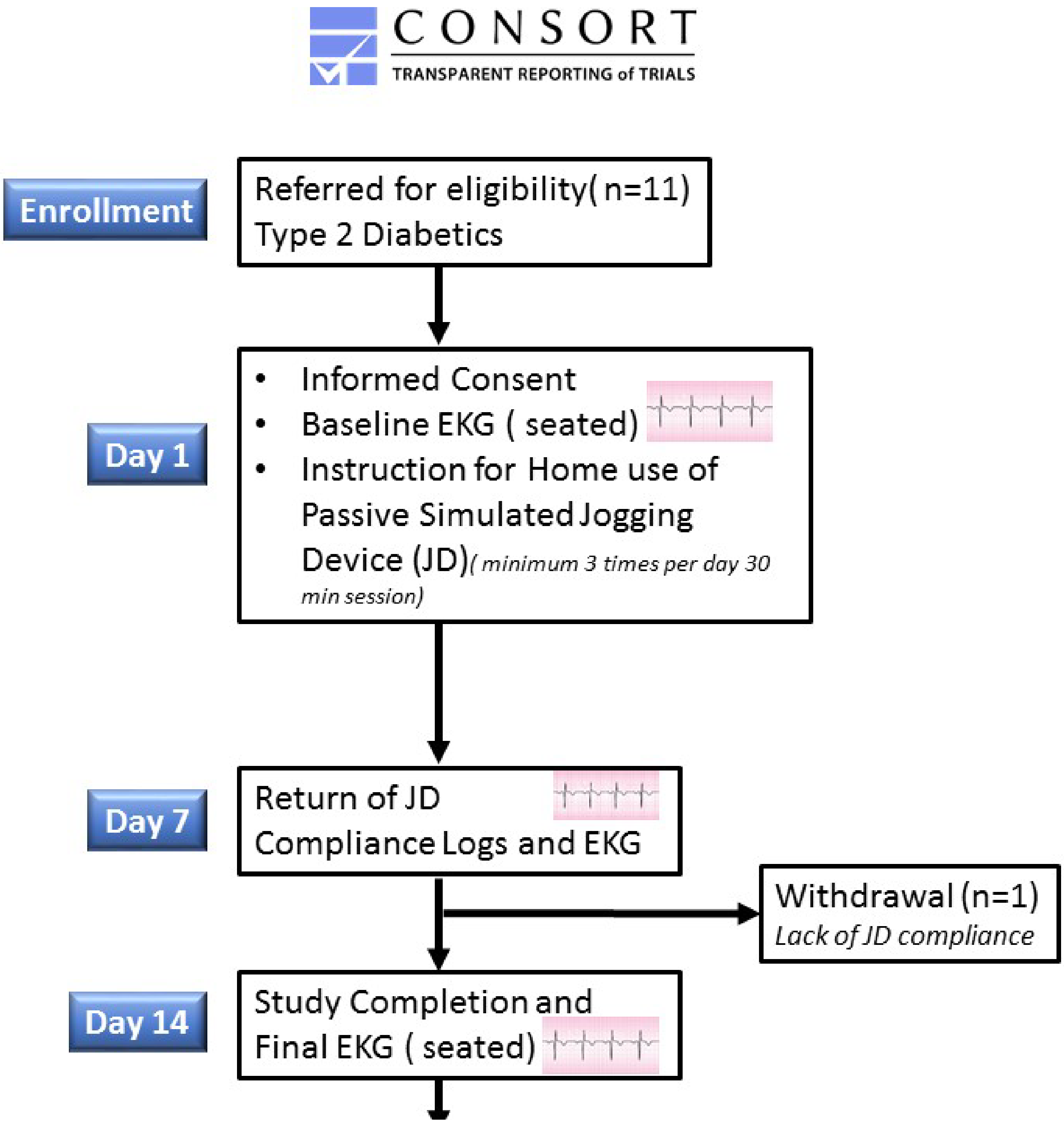
CONSORT
TRANSPARENT REPORTING of TRIALS
Enrollment
Referred for eligibility( n=11)
Type 2 Diabetics
Day 1
Informed Consent
Baseline EKG ( seated)
Instruction for Home use of Passive Simulated Jogging Device (JD)( minimum 3 times per day 30 min session)
Day 7
Return of JD
Compliance Logs and EKG
Withdrawal (n=1)
Lack of JD compliance
Day 14
Study Completion and
Final EKG ( seated)
