# Supplementary material for: A Nonrandomized Trial of the Effects of Passive Simulated Jogging on Short-Term Heart Rate Variability in Type 2 Diabetic Subjects
Source: J Diabetes Res. 2023 Apr 11;2023:4454396. doi: 10.1155/2023/4454396 (PMC10113059; doi:10.1155/2023/4454396)
Supplement: Supplementary Materials — ESM_File _S1: CONSORT checklist, study diagram, and tables. This file contains the Consolidated Standards of Reporting Trials (CONSORT) checklist and a study diagram. Table 1S contains a summary of additional study participant demographics, physical activity levels, and hemoglobin A1c. Tables 2S and 3S are the effects of age and gender on the heart rate variability changes induced by the jogging device (JD). Table 4S shows the differences between 7 days of jogging device (JD7) and 7 days after completion of JD (Post-JD) and baseline (BL) for parameters of the HRV for the entire study group. [file 4454396.f1.zip › Supplemental Material File Narrative 8.27.22.docx]

# Supplemental Material File

This file contains: Table 1S, additional demographic information of the participants including; race/ethnicity, self-reported physical activity levels, and the most recent hemoglobin A1c prior to enrollment in the study. Tables 2S and 3S, show the effects of age and gender on the heart rate variability changes induced by the jogging device (JD). Tables 2S are “The Effects of Age on JD Induced Changes in HRV” and Table 3S are “The Effects of Gender on JD Induced Changes in HRV”. Table 4S “Differences (delta after-before) for HRV parameters for the entire group “. The file also contains the Consolidated Standards of Reporting Trials (CONSORT) Study Flow Diagram and Check list.
